# Topics of the Month

**Published:** 1894-04

**Authors:** 


### TOPICS OF THE MONTH.

THE Board of Medical Examiners of the State of North Carolina is doing a most excellent work, as appears by a record thereof, prepared by Dr. Francis M. Duffy, of Newburg, embracing a period from 1885 to 1890, and published in the February number of the *North Carolina Medical Journal.* The following editorial comment thereon in the same issue of the journal possesses sufficient interest to warrant republication :

Those states which have not done so must, in the near future, defend their citizens from the quackery, charlatanry and ignorance that have gotten to be so rife in the land. As water flows to, and settles in, the lowest places, forming bogs unpleasant to the eye and dangerous to health, so will these fellows, who cannot find an abode in those states which require a demonstration of their fitness to deal with the lives of men, drift into those other states which have left their doors wide open to them, and will ply their nefarious practices to the discredit and detriment of those states.

With strict laws in North Carolina, Tennessee, Alabama and Florida, these fellows, like pus, make towards the point of least resistance, and escape into South Carolina and Georgia. All honor to the Old North state, which was the first to move in this salutary reform which forced the medical schools to require a higher standard for their graduates ; and all praise to her boards of medical examiners, who have so faithfully performed their duties and protected the lives of her people from the danger of ignorant men, who were ever ready to take advantage of their necessity and credulity.

IN AN editorial the *Medical News* recently discussed the cost of sanitary negligence. In a table furnished by the secretary of the Maryland Board of Health, it is shown that the death-rate of London is 19.11 per 1,000, while that of New York is 26.47. Following out this computation, it is shown that New York is criminally chargeable with 4,171 additional and unnecessary deaths each year as compared with Brooklyn ; 4,071 more deaths to her debt than Philadelphia and 6,774 more than Chicago. It is further affirmed that if London cared as little for the lives of her citizens as apparently does New York, there would die in the former city every year 21,524 more people than at present die. These are striking figures and rank among that class of statistics that do not misrepresent the facts. They furnish a sad commentary on the so-called economy of municipal authorities in relation to the money supply to health boards. Municipal legislatures, almost without exception, treat the estimates of health officers either with contempt or with a niggardliness that borders on criminality.

---

The death of a Polish woman, through the ignorance and neglect of a midwife, as reported in the newspapers on March 16, 1894, is a sad commentary on the intelligence of the people in this city. It is amazing that a woman should be allowed to go thirty hours after child-birth without delivery of the placenta, and finally die from this inhuman neglect in the midst of a populous and intelligent city. If it be true that the so-called midwife, who attended Mrs. Waligorska, had been pursuing her calling for two years without license, it is high time that the whole abomination relating to the present system of licensing midwives should be investigated. We recommend the committee of hygiene of the Medical Society of the County of Erie to probe this matter thoroughly. It is quite time for the state to assume the responsibility for the licensing of midwives, and thus relieve the local authorities of an unpleasant duty. We hope the bill proposed on this subject by the Medical Society of the County of New York will become a law.

---

It is one of the healthy signs of the times to see an activity springing up in all directions looking toward the prevention of the spread of consumption. It is announced among other things, that all passenger trains between Buda-Pesth and Gleichenberg, during the season at the latter place, will have special cars attached for phthisical patients. These are to be carefully renovated and disinfected after each trip. In this connection we note that Mr. Benjamin A. Parker, of Bedford, Mass., a consumptive, en route home from Yuma, Arizona, whither he had been for his health, died in a sleeping car in this city on Sunday, March 18, 1894. The car was detached from the train at Buffalo, and we hope that it was thoroughly disinfected under the direction of the health department.

THE first annual report of the Children's Hospital of Buffalo, ending October, 1893, is just at hand. It is a neat little duodecimo brochure, and tells the story of the work done for the year in a simple and entertaining fashion. The hospital was established in September, 1892, through the generosity of Mrs. Gibson T. Williams and Miss Martha Williams, who purchased the property, 219 Bryant street, and after refitting it for the purposes of a hospital, offered it, rent free, to the board of managers. During the year fifty-five patients were admitted to the hospital, forty-four of which were surgical cases, and eleven were medical in character; thirty-seven patients have been discharged and two have died in the hospital during the year, while thirty-one surgical operations have been performed during this period. The receipts for the year have been $11,183.78 ; the expenditures, $8,928.74 ; leaving a cash balance October 2, 1893, of $2,255.04. This is a creditable exhibition of the energy and executive capacity of a group of philanthropic Buffalo women.

---

THE lessons to be learned from epidemics are always many and interesting. The present waterborne fever epidemic in Buffalo accentuates the necessity in a most striking manner of subordinating the Water Department to the Health Department. No changes in the method of the water service, after it has once been established by the health department, should ever be made without the full knowledge and consent of the latter bureau. It is idle to talk about the causation of the present epidemic, call the disease by what name you will, as being other than due to polluted water.

---

THE following able exposition of the relationship of the medical profession to journalistic literature is worthy of preservation, and we commend it to the thoughtful perusal of every physician. It is taken from the editorial columns of the *Kansas Medical Journal*, issue of March 3, 1894, and we make no apology for reproducing entire such an able setting forth of so important a subject:

MEDICAL JOURNALS AND THEIR RELATION TO THE PROFESSION.

"I have no time to read medical journals," or, "I take more journals than I can read," are expressions altogether too common among physicians all over the country. It is not by any means confined to those most busily occupied by professional duties ; on the other hand, we find those physicians who have established reputations by their faithful, conscientious attention to business, and by their persistent, unrelenting labor of investigation, are the readers of medical journals. They never complain of having too much literature, but their journals are carefully laid away where at a moment's leisure they may be easily found. It is certainly no credit to any man's ability, and no guarantee of a successful or lucrative practice, to see a pile of medical journals upon his table with the wrappers still on them. It is almost an unfailing evidence of decline when a physician complains that he is too busy to keep posted. He won't be busy very long if such be the case.

There are times in every physician's history when his leisure moments from active practice are few and far between, but we are sufficiently familiar with the profession to know that this is not a perpetual condition even with the busiest of our brethren. The members of the medical profession, who are renowned for their successful work, who do not limit their practice either by county or state lines, are many of them authors of voluminous works, contributors to numerous journals and systematic readers of scores of the same. What are we to say, then, of the man who has no time to read four or five journals? Simply this: He is either not in touch with the progressive spirit of the age; he completed his knowledge of medicine in the lecture room, and sees no necessity for further investigation; or he is too ignorant of the principles of the science which he pretends to practise to understand or appreciate the thoughts of labors of his more intelligent brothers.

It is not a waste of time to read even the poorest of the medical journals.

We receive each month more than 150 journals which it is our business to examine. Some of these are placed high in the rank of medical journalism, others are suffering for lack of appreciation and support, but we often find as many practical suggestions and as many items of real value to the general practitioner among the latter class as in the former.

A man who reads to learn will find something he did not know before in almost any magazine or paper he takes up. We often get new ideas from rereading a book we supposed we were perfectly familiar with. The human mind is incapable of storing up every item of interest it lights upon. It needs to be continually fed, and often by reading something apparently stale a forgotten idea is called to mind which may be of incalculable value, but journal items are generally new. It is there that the latest discoveries, the most recent investigations, are reported to the medical world.

Medical editors are especially shy of communications having an ancient odor, and it is much for this reason that readers of numerous medical journals are better posted on recent facts in medicine than their brothers who confine themselves to text-books.

These new things in medicine do not all come from Germany, England, France, nor even New York. The physicians of the west are as much alive to the demands of the time and to the possibilities of advancement as are our more favored metropolitan brothers. Western medical journals are, therefore, the mediums of communication from the east to the west, and also from the west to the east.

---

THERE is another despicable bill before the legislature that ought to receive the condemnation of every respectable man and woman in the State of New York. We refer to the attempt to legalize the so-called Christian science cure by statutory enactment. We cannot believe that an intelligent body of men, such as composes the legislature of the State of New York can seriously contemplate favorable action on the proposed measure. The State has already committed to the Regents of the University sole supervision of the right to practise medicine within its borders, and it would not be consistent to make an exception in favor of a group of persons who practise on the weaknesses of unprotected humanity by pretending to invoke the aid of the supernatural in the cure of their physical infirmities. If it should do so, then we may expect all the other quacks and fakirs in medicine to ask for similar privileges, which, if it would be consistent, the legislature must grant. There are two serious objections to that form of chicanery, misnamed Christian science,—it is neither Christian-like nor scientific.

---

THE medical officers who served under contract during the late war, under the title of acting assistant surgeons, are petitioning congress to have commissions issued to them for their respective terms of service. This would give them the same legal standing and afford them the same privileges as are enjoyed by regular army medical officers or those of the volunteer service. We doubt the propriety of this measure, though we recognize the valuable services that these contract surgeons performed. As a rule, however, they were employed in general hospitals, and were privileged to terminate their contracts at will. Not so with the medical officers, regular or volunteer service, who were subjected to the stern rules of military discipline with the armies in the field. Wherever there has been individual injustice, however, we shall be glad to see a law passed that will correct or remove the evils.

The State of Ohio is at last in a fair way to enjoy the benefits of a separate state medical examining and licensing board. A bill creating such a body has passed one house of the legislature and seems likely to be approved by the other branch. Ohio has been for many years a camping-ground for medical tramps, quacks, fakirs and impostors, and we congratulate our colleagues of the Buckeye state upon the prospect of an early deliverance from this horde of vultures. One of the peculiarities of the Ohio bill places midwives under the supervision of the board, a feature which we commend. It is rather amusing, however, to note another feature, namely: "Nothing in this act shall be construed to prohibit advertising in newspapers."

